# Digits-in-noise test in Brazilian Portuguese: how demographic and socioeconomic variables influence normal-hearing subjects

**DOI:** 10.1590/2317-1782/20212021274

**Published:** 2022-04-26

**Authors:** Victor Vasconcelos Barros, Aryelly Dayane da Silva Nunes-Araújo, Aline Roberta Xavier da Silva, Hannalice Gottschalck Cavalcanti, Deborah Viviane Ferrari, Sheila Andreoli Balen

**Affiliations:** 1 Universidade Federal do Rio Grande do Norte – UFRN - Natal (RN), Brasil.; 2 Universidade Federal da Paraíba – UFPB - João Pessoa (PB), Brasil.; 3 Faculdade de Odontologia de Bauru, Universidade de São Paulo – USP - Bauru (SP), Brasil.

**Keywords:** Hearing, Speech in Noise, Mobile devices, Telehealth, m-health, Adults, Elderly, Educational Levels, Socioeconomic Status

## Abstract

**Purpose:**

Verify how demographic and socioeconomic variables on the in-noise speech recognition threshold (SRT) from the digits-in-noise test (DIN) in Brazilian Portuguese influence normal-hearing subjects.

**Methods:**

Cross-sectional, prospective study. The convenience sample had 151 normal-hearing subjects between 12 and 79 years (mean=34.66) who underwent pure tone audiometry and digits-in-noise test with white noise using a sequence of three numbers in diotic stimulus (in-phase) on the same day. The DIN was performed using a Motorola Z3 Play smartphone with internet access and in-ear headphones. In-noise digit speech recognition threshold (SRT) was analyzed for gender, age, educational levels, and socioeconomic status. We used the non-parametric version of the Kruskal-Wallis and Mann-Whitney U tests to compare independent samples adopting a significance level of 5%.

**Results:**

The mean SRT was -8.47 dBNA (SD -3.89) with a median of -9.6 dBNA. The SRT was proportionally inverse to educational levels and socioeconomic status and more negative (better) with lower age groups. Gender did not influence the DIN SRT.

**Conclusion:**

Age, educational levels, and socioeconomic status influenced the DIN threshold. These variables must be considered when analyzing DIN performance in Brazilian Portuguese in normal-hearing subjects.

## INTRODUCTION

In 1985, the World Health Organization estimated that 1% of the world's population had disabling hearing loss. In 2018, that estimation rose to 6.1%, corresponding to 466 million people living with auditory sensory deprivation. This estimate is that 700 million people will have disabling hearing loss^(1)^ by 2050.

Hearing loss dramatically impacts people's lives, leading to changes in communication and learning that can cause social isolation, emotional problems, and low academic and professional performance^(2)^. Besides, untreated hearing loss generates a high annual cost worldwide, ranging from around US$ 981 billion^(3)^. Therefore, early detection and intervention are essential to lessening hearing loss impacts. Therefore, measures for hearing screening should be established at different stages of life, including neonates and infants, preschool-age children, and adults — especially older adults and other populations at greater risk due to exposure to noise, chemicals, and ototoxic medications^(1)^.

Increasingly validated and reliable technologies need to be implemented to detect hearing loss as its prevalence grows. In addition, economic issues, easy-to-apply technologies, accessibility, and timeliness are essential. Therefore, hearing screening instruments^(4)^ must be validated for the appropriate populations that allow identifying hearing loss.

There is an exponential growth of mobile technologies transforming different aspects of society, including health care. It is a significant opportunity to make available several technical solutions. There are 346 million mobile devices in Brazil, 83% of which are cell phones, even though there is still inequality in distribution^(5)^.

The digits-in-noise test (DIN) has become increasingly known among hearing screening procedures. The DIN was initially proposed in the Netherlands as an automated hearing screening using a landline phone^(6)^. It plays three-digit sequences (0-9) mixed with noise. The speech stimulus presentation level is fixed, and the masking noise level is adaptively increased or decreased until there is a signal/noise ratio in which individuals can correctly recognize 50% of the presented stimuli. That level is the speech recognition threshold (SRT)^(6,7)^.

In the DIN, groups of three digits are played diotically, that is, in both ears simultaneously with white noise. That is different from the dichotic digits test (DDT) used to assess central auditory processing in which two pairs of digits are presented simultaneously in both ears in a dichotic situation. Thus, the DIN and the DDT are distinct in stimulus presentation and purpose.

The DIN uses speech material (digits) with low linguistic demand in a closed set^(7)^, making it suitable for populations with different language skills. Besides, it is similar to daily life hearing situations. Finally, as it is an automated procedure, users can perform it in minutes^(7)^. The DIN has been translated into several languages such as English, Flemish, Finnish, Australian English, Turkish, South African English, Swedish, Chinese, and Greek^(8)^.

It was adapted for mobile device^(7,8)^ administration in 2016 with a structure similar to the original proposal as these devices make it possible to provide users with a more user-friendly test. Additionally, the audio signal is broadband and digital, unlike a landline telephone whose bandwidth is approximately 300 to 3,400 Hz^(7,8)^.

Screening methods that make it easier for populations to access information related to hearing health and reduce the demands for assistance in health care units have been the foundation for several pieces of research in both the academic environment and tech field. These methods can be used to identify hearing loss, reduce expenses, and facilitate monitoring and early detection^(9)^.

Its application using smartphones' headphones compared to headphones traditionally attached to audiometers showed the same results^(10)^, even in the DIN Portuguese version.

It is a tool with great potential for hearing screening as it simulates everyday listening situations in an adaptive test^(11)^ which contributes to its wide use and widespread access to a simple screening instrument due to its low cost and high sensitivity^(12)^. The DIN was recently translated and adapted into Brazilian Portuguese^(10,12,13)^. The World Health Organization has indicated it for hearing screening due to its features, and it is available for download in app stores as “HearWHO App.”^(14)^

A scoping review compiled 39 studies which were selected out of 95 studies related to the “digit triplet test” and “digits in noise.” The authors discuss several variables between the studies, such as test language, speech and noise material, platform, procedural aspects, stimulation method, speech/noise adaptation, measurement procedure, aspects of validity, and reliability. They discovered that studies with the DIN in the last fifteen years have shown that the test is highly reliable and efficient to measure functional hearing loss and estimate hearing loss in different age groups and populations^(8)^.

Different countries have intrinsic test changes^(8)^. A DIN study conducted in English in South Africa showed that English competence affected the auditory performance of subjects in 11 different languages.

A preliminary accuracy study in Brazil using the DIN found that factors external to the test and intrinsic to subjects, such as age, education, and socioeconomic status, may influence results^(12)^, which means there might be a connection to social determinants of health (SDH). SDH express that individuals and population groups' living and working conditions are related to their health situation, including social, economic, cultural, ethnic/racial, psychological, and behavioral factors^(15)^, which shows a connection between hearing and SDH. Studies performed in China and the United States have shown relationships between hearing loss and lower socioeconomic and educational levels^(16,17)^. The World Health Organization's World Hearing Report (2021) also portrays that reality^(18)^. 

The analysis of the influence of social determinants of health can contribute so that in the validation process of new tests, including hearing screening, these variables are considered at the cut-off points to adapt the test methodology to each country's social reality. Unfortunately, the scope review^(8)^ did not find studies with those specific objectives, which shows limited literature on the impact of educational and socioeconomic statuses on the DIN SRT. As the DIN in Brazilian Portuguese is currently undergoing validation, it is essential to know its results against demographic and socioeconomic variables in normal-hearing subjects so that they can be exempt from the influence of type, degree, and configuration of hearing loss in hearing-impaired subjects, to whom the DIN is intended.

Consequently, this study aims to analyze the influence of demographic and socioeconomic variables on the DIN in Brazilian Portuguese on normal-hearing subjects.

## METHODS

This prospective multi center cross-sectional study was approved by the Hospital Universitário Onofre Lopes's Human Research Ethics Committee (protocol No. 2525183). All participants or guardians signed an informed consent or assent form, and subjects between 12 and 18 years old signed the assent form.

This study sample had 151 normal-hearing subjects between 12 and 79 years old (34.66±16.17). The subjects were from the Brazilian states of Rio Grande do Norte and Paraiba and underwent pure-tone audiometry, tympanometry, and the digits-in-noise test subsequently on the same day. Subjects from Paraíba were recruited and assessed at the Hospital da Universidade Federal da Paraíba, and those from Rio Grande do Norte attended a hearing health service provider accredited to the Sistema Unico de Saúde (SUS) through convenience samples between September 2018 and March 2020. The teams performing the DIN and pure-tone audiometry in both centers were trained and followed the same collection procedures. They were both in a multi-center study between Universidade Federal do Rio Grande do Norte, Hospital da Universidade Federal da Paraíba, Faculdade de Odontologia da Universidade de São Paulo e University of Pretoria, and South African company hearX. The researchers recruited the subjects in the waiting room of the hearing health service provider.

Study participants met the following inclusion criteria: Subjects aged 12 years or older who underwent pure-tone audiometry and could identify the graphic representation of the digits 0 to 9 with no motor or uncorrected visual impairment, as well as cognitive deficit or known neurological disorders. Additionally, they presented a four-tone average of up to 25 dB HL in the pure-tone audiometry in both ears and had no middle ear alterations confirmed by tympanometry with peak pressure between +100 to -100 daPA and compliance greater than 0.3 cc. Subjects were considered normal hearing according to this criterion.

A Motorola Z3 Play smartphone with internet access performed the test version of the DIN. That preliminary version of the application was developed for Brazilian research. Original smartphone in-ear headphones were used for the procedure with the original adapter connecting the headphones to the smartphone. A preliminary study showed no difference in in-noise digit speech recognition threshold (SRT) of the DIN between the in-ear headphones and the TDH-3910^(10)^. The researchers first registered subjects in the test version of the DIN. In a non-acoustically treated quiet environment, all subjects were instructed to listen to three-digit sequences mixed with white noise played simultaneously and then click the numbers they would hear using the smartphone. If they did not hear any of the digits, they were instructed to assume a sequence of digits to proceed. After instructions and before starting the test, the subjects were informed that they could adjust the test volume according to the preliminary version of the application to ensure that they could hear the numbers clearly and comfortably. That was the only volume adjustment performed by the user as it also occurs in the final version of the application available in other languages. The device volume was fixed at its maximum level.

The test randomly showed 23 three-digit sequences (0-9) in white noise at 70 dB SPL in a fixed signal-to-noise ratio between -20 dB to 20 dB in diotic mode (in-phase). The sequences were initially presented at a signal/noise ratio of 0 dB and varied according to the patient's responses. The researcher did not interfere. The first three presentations of each stimulus were considered training and thus disregarded from the final result. The subjects were instructed to type the three-digit sequence that they had heard with the noise on the smartphone's virtual keyboard. The following speech signal and noise had a lower signal-to-noise ratio if they selected the correct sequence. However, if they selected the wrong sequence, the software would increase the signal-to-noise ratio the next time, with a dB variation in both cases. After the 23 sequences, the software generated the digit recognition threshold (SRT) in noise using the average signal-to-noise ratio between sequences 4 and 23. The final screen displayed a number representing the SRT value. In addition, the application would download a table with each subject's presentation's detailed results. This data was then tabulated in an Excel database. More negative DIN SRT values are expected in normal-hearing subjects, which means better performance in the test.

Pure-tone audiometry was performed in a soundproof booth with an AD229e calibrated audiometer to determine the audiological status. Tested frequencies ranged from 250 Hz to 8,000 Hz in the airway and 500 Hz to 4,000 Hz in the bone pathway when patients had a minimal response in any of these frequencies at an intensity greater than 25 dB HL in the airway test. Those with four-tone mean auditory thresholds (500 Hz, 1,000 Hz, 2,000 Hz, and 4,000 Hz) up to 25 dB HL in both ears were classified as normal-hearing^(19)^.

Data were collected independently by the speech-language pathologists who performed the pure-tone audiometry and those who applied the DIN. As a result, the team only learned the results of each procedure during data tabulation.

In addition to the audiological tests, all subjects orally answered a questionnaire to collect the following demographic variables: Age and gender, and the socioeconomic variables educational levels and socioeconomic status.

The sample was subdivided into the following age groups: G1: 12-19, G2: 20-39, G3: 40-59, G4: 60-79 years old.

Educational levels were split into three levels: “Low” for subjects who declared themselves illiterate or who had not completed elementary school, corresponding to 0-9 years of school time; “Average” for subjects who completed elementary or high school, equivalent to 9-16 years of school time; and “High” for those with incomplete or complete higher education, totaling more than 16 years of school time.

The Associação Brasileira de Empresas de Pesquisa (ABEP)^(20)^ questionnaire was applied to assess socioeconomic status. The document is divided into two categories. The first includes which and how many “items” participants have at home, including bathrooms, computers, dishwashers, automobiles, microwave ovens, among others. The second category is the family's provider's educational level (school years) and access to public services (piped water supply and paved streets). Therefore, this study was divided into three socioeconomic statuses based on the analysis proposed by ABEP^(20)^: “low” for subjects scoring 1-16 points; “medium” for 17-28 points; “high” for 29-100 points.

The Kolmogorov-Smirnov test was used to check for distribution normality during data analysis. As normal distribution was not found, the non-parametric version of the Kruskal-Wallis and Mann-Whitney U tests were used to compare the independent variables (gender, age group, educational level, and socioeconomic status) against the dependent variable, which was the DIN SRT value. A significance level of 5% was adopted.

## RESULTS

This study sample had 151 normal-hearing subjects between 12 and 79 years old (34.66±16.17). The DIN SRT of the total sample of normal hearing subjects had a median of -9.6 dB, a minimum value of -13 dB and a maximum value of +1.8 dB of the DIN SRT.

Table 1 shows the sample distribution as a function of demographic and socioeconomic variables and the DIN SRT's descriptive and inferential statistics regarding these variables.

**Table 1:** DIN SRT descriptive and inferential statistics regarding demographic and socioeconomic variables of the study sample

		**Diotic DIN SRT**							**p – value**
		**N (%)**	**Mean (SD)**	**Min**	**Max**	**Q25**	**Med**	**Q75**	
Gender	Male	42 (27.82%)	-9.13 (2.84)	-13.00	-1.00	-11.20	-9.80	-7.40	.404
	Female	109 (72.18%)	-8.52 (3.512)	-12.60	5.20	-11.10	-9.40	-7.40	
Age group	12-19 (G1)	15 (9.94%)	-8.86 (3.87)	-12.60	1.80	-11.60	-10.20	-7.40	^**^
	20-39 (G2)	83 (54.97%)	-9.71 (2.33)	-13.00	-2.00	--11.40	-8.40	-6.68	
	40-59 (G3)	41 (27.15%)	-7.34 (3.91)	-11.60	5.20	-10.50	-8.00	-5.60	
	60-79 (G4)	12 (7.94%)	-6.00 (3.78)	-10.20	2.00	-8.40	-8.00	-3.20	
Education	Low	31 (20.53%)	-6.26 (4.17)	-11.40	3.60	-9.20	-7.40	-3.60	.001^*^
	Medium	38 (25.17%)	-8.47 (3.24)	-11.80	5.20	-10.80	-9.20	-7.40	
	High	82 (54.30%)	-9.71 (2.47)	-13.00	-2.00	-11.60	-10.20	-8.40	
SES	Low	12 (7.94%)	-4.83 (5.04)	-10.80	3.6	-8.90	-6.10	0.20	.001*
	Medium	44 (29.14%)	-7.79 (3.53)	-11.80	5.2	-10.50	-8.50	-6.50	
	High	95 (62.92%)	-9.59 (2.45)	-13.00	--2,00	-11.40	-10.20	-8.20	

* ρ-value <0.05 significant. Kruskal Wallis and Mann-Whitney U tests;

** p-value>0.05 (groups G1XG2, G1XG3, and G3XG4) and p<0.05 (G1XG4, G2XG3, G2XG4);

N: Número de sujeitos (%); **Caption:** SRT – in-noise digit recognition threshold Q25 – 25% quartile. Q75 – 75% quartile. N = sample N; % = percentage. SES – socioeconomic status

All variables but gender influenced the DIN SRT result. As for age groups, the youngest group (12-19 years old) had a significantly better DIN SRT only when compared to older adults (60-79 years old). On the other hand, adults between 20 and 39 years old had better DIN SRT when compared to adults between 40 and 59 years old and older adults over 60 years old.

## DISCUSSION

This study found that the DIN SRT in Brazilian Portuguese in normal-hearing subjects is related to educational level, socioeconomic status, and age group. Gender did not influence the DIN SRT.

### DIN SRT in normal-hearing subjects

The average of -8.47 dB in the DIN SRT in diotic condition (in-phase) found in normal-hearing subjects in this study is higher than the average Potgieter et al.^(7)^ reported in normal-hearing subjects (-10.7 dB for both ears < 15 dB four-tone mean). However, it is similar to the cut-off value these authors found of -8.4 dB for the best ear or -8.9 dB for both ears with normal hearing (<15dB four-tone average).

The expected results are similar to the average DIN SRT of subjects who do not speak English (-8.7 dB)^(11)^. On the other hand, people with better English skills or native speakers had the average DIN SRT at -10.4 and -10.2 dB, respectively.

The digit test assessment proved reliable in groups of normal-hearing subjects and people with hearing aids and cochlear implants. It is a tool for hearing screening and evaluating the rehabilitation process^(20,21)^, as identified in this sample. However, due to the influence of socioeconomic and demographic variables, the SRT result was not similar to the findings in the full version of the test.

### Gender

The studied sample had 72.18% of female subjects, although this predominance over male subjects was not evidenced by the difference between the results of the DIN SRT between genders. This variable was not a predictor of DIN SRT results in a study with English SRT with varied English competencies and speakers of different languages in South Africa^(11)^.

### Age

Subjects over 60 years old had worse diotic DIN SRT when compared to younger subjects (12-39 years old). That result is different from another study with SRT results similar to those of young people starting at 12 years old^(22)^. A similar result was found in the study with DIN in Brazilian Portuguese with normal-hearing children without auditory processing disorders and with auditory processing disorder aged between 8 and 11 years^(13)^. The SRT values for these two groups had means similar to those found in another study with a digits-in-noise test in Brazilian Portuguese^(12)^. That may indicate the impact of the sociodemographic differences, something typical to the sample since these studies' populations come from the same region despite having different age groups.

A retrospective study with 24,072 subjects found that age impacts DIN results. That may be due to older adults' decreased cognition and difficulty understanding explanations^(23)^. Even though these are minor difficulties, they may pose a challenge and inconvenience for this age group when interacting with their smartphones^(24)^. Researchers addressed the need to consider age when determining test results as their accuracy may differ based on the age group. These authors showed that age could be a significant predictor of the DIN SRT for hearing subjects with better ears MQ≤25 dB HL^(25)^.

Similar information can be found in a study that found decreased cognitive aspects and auditory processing skills with other in-noise speech recognition tasks as subjects aged^(26)^.

Thus, considering age when determining the RDT result in normal-hearing subjects is essential as it may contribute to screening test accuracy^(27)^. Additionally, other studies may consider a cognitive screening to identify how these potential changes may impact DIN results.

### Education

Although most subjects in this study had high educational levels, subjects with higher education levels tend to have better speech understanding in noise with better SRT.

Different researchers also realized that the difference in educational level is related to hearing difficulties for auditory processing activities. Among their hypotheses is the difference in the history of the older adults who had precarious access to education^(27)^.

Speech understanding in noise is also impaired in auditory processing activities due to a lower educational level. It is up to discussion whether that result is influenced by working memory in addition to auditory factors, although that feature is significant in other auditory processing skills but not in the speech-in-noise test^(28)^.

### Socioeconomic status

Socioeconomic status considers education, occupation, and income; therefore, socioeconomic and educational level variables addressed in this study are directly related. The statistical analysis in this study shows that equivalence. There is a statistically significant difference between low, medium, and high groups for educational level and socioeconomic status. That is, the higher the socioeconomic status and educational level, the more negative the DIN SRT value.

Socioeconomic status is intrinsically related to health, so higher-income people are usually healthier than those with lower socioeconomic statuses^(29)^. Among the world population with hearing loss, there is a higher prevalence in low- and middle-income countries in addition to low health care service capacity^(1)^. Thus, the prevalence of this disorder changes according to the region^(30)^, and the socioeconomic component is one of its determining factors.

Socioeconomic and educational factors can be related to individuals' audiological status. For example, a study with 3379 people in the United States showed a direct correlation between socioeconomic status, educational level and hearing loss, showing that this impairment may be a factor or a product of socioeconomic status. In addition, research revealed associations between unemployment, age, education, gender, and hearing status. As for the DIN, individuals with normal hearing (confirmed through pure-tone audiometry) generally did not perform appropriately for their audiological status^(16)^.

Besides health promotion and prevention methods through digital technologies, professionals should always consider the influence of aging on the DIN, especially with older adults, due to their physical restrictions and difficulty in handling electronic devices. Still, the DIN is likely to target the younger population that, despite its lower prevalence of hearing loss^(23)^, has caused a worldwide concern given their excessive use of individual music devices used with loud volumes.

Therefore, it would be ideal to consider the different factors studied in the DIN result, adopting different cut-off points according to the individual's socioeconomic status, educational level or age group.

### Study limitations

When analyzing factors that may have changed search results, the available test version used for testing digits in noise stands out. Its access through an online link made it impossible to use when there was no internet connection. As its response to touchscreens is still not fully deployed, the researcher needed to intervene sometimes to zoom out after users accidentally double-tapping their devices. User experience could be improved through the final app version.

Different age groups, educational level and socioeconomic status characteristics, and other Northeastern Brazilian population features could also impact the results, which is why we suggest further studies in different Brazilian regions.

### Perspectives for future research

Further studies must check the finished app version against its trial version. Besides, future research should consider analyzing demographic and socioeconomic variables studied at the test cut-off point to achieve better accuracy in the digits-in-noise test in Portuguese, considering population characteristics and favoring its large-scale application as a tool to identify hearing loss.

## CONCLUSION

The diotic digits-in-noise test in Brazilian Portuguese is associated with extrinsic variables, showing better responses in people between 20 and 39 years of age with high socioeconomic status and educational levels. Gender did not influence digit recognition in the DIN.
