# ERRATUM: Digits-in-noise test in Brazilian Portuguese: how demographic and socioeconomic variables influence normal-hearing subjects

**DOI:** 10.1590/2317-1782/20212022110

**Published:** 2022-06-27

Due to technical problems during the editorial production of the article “Digits-in-noise test in Brazilian Portuguese: how demographic and socioeconomic variables influence normal-hearing subjects” (DOI https://doi.org/10.1590/2317-1782/20212021274), published in CoDAS, 2022;34(6):e20210274, was published with an error that caused the omission of the acknowledgments text from the article.

On page 6, where the text ends and the list of bibliographic references begins, it should read:

**ACKNOWLEDGEMENTS**

The authors would like to thank researchers De Wet Swanepoel and Cas Smits for their contributions and partnership in developing the Brazilian Portuguese version of the digits-in-noise test.

The publisher apologizes for the errors.

