# Supplementary material for: Digits-in-noise test in Brazilian Portuguese: how demographic and socioeconomic variables influence normal-hearing subjects
Source: Codas. 2022 Apr 26;34(6):e20210274. doi: 10.1590/2317-1782/20212021274 (PMC9886289; doi:10.1590/2317-1782/20212021274)
Supplement: Translation

# Translation: Teste de dígitos no ruído no Português Brasileiro: influência das variáveis demográficas e socioeconômicas em normo-ouvintes

**Authors:** Victor Vasconcelos Barros, Aryelly Dayane da Silva Nunes-Araújo, Aline Roberta Xavier da Silva, Hannalice Gottschalck Cavalcanti, Deborah Viviane Ferrari, Sheila Andreoli Balen

**Affiliations:** 1 Universidade Federal do Rio Grande do Norte – UFRN - Natal (RN), Brasil.; 2 Universidade Federal da Paraíba – UFPB - João Pessoa (PB), Brasil.; 3 Faculdade de Odontologia de Bauru, Universidade de São Paulo – USP - Bauru (SP), Brasil.

**Keywords:** Audição, Fala no Ruído, Dispositivos Móveis, Telessaúde, m-saúde, Adultos, Idoso, Escolaridade, Nível Socioeconômico

## Abstract

**Objetivo:**

Verificar a influência das variáveis demográficas e socioeconômicas no limiar de reconhecimento de fala no ruído (LRF) obtidos no teste de dígitos no ruído (TDR) no Português Brasileiro em normo-ouvintes.

**Método:**

Estudo transversal e prospectivo. A amostra de conveniência foi composta por 151 sujeitos normo-ouvintes com idade entre 12 e 79 anos (média =34,66) que realizaram audiometria tonal liminar e teste de dígitos no ruído branco com sequência de trios numéricos em estímulo diótico (inphase) no mesmo dia. O TDR foi realizado com um smartphone Motorola Z3 play com acesso à internet com fones de ouvido intra-auriculares. Os limiares de reconhecimento dos dígitos no ruído (LRF) foram analisados em função do sexo, idade, escolaridade e nível socioeconômico. Foi utilizado para comparar as amostras independentes, o teste não-paramétrico Kruskal-Wallis e Mann-Whitney, adotando-se o nível de significância de 5%.

**Resultados:**

A média do LRF foi de -8,47 dBNA (dp -3,89), com mediana de -9,6 dBNA. O LRF foi proporcionalmente inverso à escolaridade e nível socioeconômico e mais negativo (isto é, melhor) com menor faixa-etária. Não houve evidência de influência do sexo no LRF do TDR.

**Conclusão:**

Idade, escolaridade e nível socioeconômico mostraram influenciar o limiar no TDR; essas variáveis devem ser consideradas na análise de desempenho do TDR no Português Brasileiro em sujeitos normo-ouvintes.

## INTRODUÇÃO

Em 1985, a Organização Mundial de Saúde estimava que 1% da população mundial possuía perda auditiva incapacitante. Em 2018, a estimativa subiu para 6,1%, o que corresponde a 466 milhões de pessoas vivendo com privação sensorial auditiva. A projeção dessa estimativa é que até 2050, 700 milhões de pessoas tenham perda auditiva incapacitante^(1)^.

A perda auditiva possui diversos impactos sobre a vida de uma pessoa, levando, de maneira geral, a alterações na comunicação e na aprendizagem e assim podendo conduzir ao isolamento social, problemas emocionais, baixo desempenho acadêmico e/ou profissional^(2)^. Além disso, perdas auditivas não tratadas geram no mundo todo um alto custo anual, numa faixa de 981 bilhões de dólares^(3)^. A fim de diminuir os impactos da deficiência auditiva, a detecção e intervenção precoce são necessárias. Portanto, medidas para a triagem auditiva devem ser estabelecidas nos diferentes estágios de vida, incluindo neonatos e bebês, crianças em idade pré-escolar, adultos - especialmente os mais velhos e outras populações que apresentam maior risco em função de exposição a ruído, produtos químicos e medicamentos ototóxicos^(1)^.

É necessária uma implementação de tecnologias cada vez mais válidas e confiáveis para detectar perdas auditivas na medida que a sua prevalência cresce. Questões econômicas, tecnologias de fácil aplicação, acessibilidade e rapidez são essenciais para isso. Portanto é necessário a utilização de instrumentos de rastreio auditivo^(4)^ validados para as populações em que serão aplicados, permitindo a identificação da perda auditiva.

Há um crescimento exponencial das tecnologias móveis que vem transformando diferentes aspectos da sociedade, incluindo a área da saúde. Isso representa uma importante oportunidade para disseminação de variadas soluções desta natureza. No Brasil existem 346 milhões de dispositivos móveis, sendo 83% destes celulares, embora ainda exista desigualdade de distribuição^(5)^.

Dentre os procedimentos para a triagem auditiva, o teste de dígitos no ruído (TDR) vem ganhando notoriedade. O TDR foi proposto inicialmente na Holanda, como forma de triagem auditiva automatizada, por meio de telefone fixo^(6)^. Neste teste, sequências de três dígitos (0 a 9) são apresentadas na presença de ruído. O nível de apresentação do estímulo de fala é fixo e o nível do ruído mascarante é adaptativamente aumentado ou diminuído, até que se obtenha a relação sinal/ruído em que o indivíduo conseguiu reconhecer corretamente 50% dos estímulos apresentados. Este nível é chamado limiar de reconhecimento de fala (LRF)^(6,7)^.

Destaca-se que o TDR é um teste no qual os trios de dígitos são apresentados dioticamente, isto é, nas duas orelhas simultaneamente com o ruído branco, sendo diferente do Teste dicótico de dígitos (TDD) utilizado na avaliação do processamento auditivo central no qual são apresentados dois pares de dígitos simultaneamente nas duas orelhas em situação dicótica. Desta forma, tanto na apresentação dos estímulos quanto na sua finalidade o TDR e o TDD são distintos.

O TDR utiliza um material de fala (dígitos) com baixa demanda linguística e apresentado em conjunto fechado^(7)^, fazendo com que seja adequado para populações com distintas competências de linguagem. Além disso, o teste se aproxima de situações de escuta reais. Finalmente, por se tratar de um procedimento automatizado, pode ser realizado em questões de minutos pelo próprio usuário^(7)^. Este teste foi traduzido para diversos idiomas como inglês, flamengo, finlandês, australiano-inglês, turco, inglês sul-africano, sueco, chinês e grego^(8)^.

A partir de 2016, o TDR foi adaptado para administração via dispositivos móveis^(7,8)^. com estrutura semelhante à proposta à original. O uso destes dispositivos possibilita fornecer ao usuário um teste mais amigável e, além disto, o sinal de áudio produzido é de banda larga e qualidade digital, diferentemente do telefone fixo cuja largura de banda é de aproximadamente 300 a 3400 Hz^(7,8)^

Métodos de triagem que facilitam o acesso da população a informações que se referem à saúde auditiva e diminuem as demandas de atendimentos nas Unidades de Saúde têm sido base para diversas pesquisas no meio acadêmico e na área tecnológica. Isso se deve ao fato de que esses métodos podem ser usados como ferramentas na identificação da perda auditiva, reduzindo gastos e facilitando o monitoramento e detecção precoce da mesma^(9)^.

Sua aplicação com uso de fones de ouvido do próprio *smartphone* em comparativo a fones que são tradicionalmente acoplados aos audiômetros apresentaram os mesmos resultados^(10)^, inclusive na versão em Português do TDR^(10)^.

É uma ferramenta com alto potencial para triagem auditiva, pois simula situações de escuta cotidiana em teste adaptativo^(11)^, contribuindo para uma ampla utilização, permitindo, à população, o acesso a um instrumento de triagem simples, por possuir baixo custo e alta sensibilidade^(12)^. O TDR foi recentemente traduzido e vem sendo adaptado para o português brasileiro^(10,12,13)^. Por suas características, é indicado para rastreio auditivo pela Organização Mundial de Saúde e disponibilizado nas plataformas de *download* com o título *HearWHO App* ^(14)^.

Em uma revisão de escopo, foi apresentada uma compilação de 39 estudos selecionados a partir da identificação de 95 estudos relacionados ao “*digit triplet test*” e/ou “*digits in noise*”. Os autores discutem sobre diversas variáveis entre os estudos como o idioma do teste, o material de fala e ruído utilizados, plataforma, aspectos procedimentais, método de estimulação, adaptação de fala/ruído, procedimento de medição, aspectos de validade e confiabilidade. Os autores, acabam por ressaltar que os estudos com o TDR, nos últimos quinze anos, evidenciaram ser este um teste altamente confiável e eficiente para medir a perda da capacidade auditiva funcional, bem como para estimar a perda auditiva de pessoas em diferentes faixas-etárias e populações^(8)^.

Ao redor do mundo, é possível observar alterações intrínsecas do teste^(8)^. Dentro de estudo com TDR em inglês na África do Sul, foi observada a influência do nível de competência em inglês no desempenho auditivo de sujeitos falantes de 11 diferentes línguas^(11)^.

Em estudo preliminar de acurácia no Brasil utilizando o TDR foi observado possível influência de fatores externos ao teste e intrínsecos ao sujeito como idade, escolaridade e nível socioeconômico^(12)^, evidenciando uma possível relação com os Determinantes Sociais da Saúde (DSS). Os DSS expressam que as condições de vida e trabalho de indivíduos e/ou grupos populacionais tem relação com a sua situação de saúde, podendo ser citados os fatores sociais, econômicos, culturais, étnicos/raciais, psicológicos e comportamentais^(15)^, evidenciando as relações entre audição e DSS. Estudos realizados na China e nos Estados Unidos evidenciaram relações da perda auditiva com menores níveis socioeconômicos e de escolaridade^(16,17)^. O relatório mundial da audição da Organização Mundial de Saúde (2021) também retrata esta realidade^(18)^. 

A análise da influência dos determinantes sociais de saúde pode contribuir para que no processo de validação de novos testes, inclusive de triagem auditiva, sejam consideradas, estas variáveis nos pontos de corte dos testes, no intuito, de adequar a metodologia do teste a realidade social do País no qual ela está sendo proposta. Infelizmente, na revisão de escopo^(8)^ não foram evidenciados estudos que tivessem estes objetivos como foco de suas investigações científicas, o que acaba por evidenciar uma literatura restrita sobre o impacto do nível de escolaridade e socioeconômico no LRF do TDR. Como o TDR na língua Portuguesa é um teste em processo de validação é de suma importância conhecer quais os seus resultados frente as variáveis demográficas e socioecômicas em normo-ouvintes para que possam estar isentas da influência do tipo, grau e configuração da perda auditiva nas pessoas com deficiência auditiva, para o qual o TDR se destina.

Diante do exposto, o objetivo deste estudo foi analisar a influência de variáveis demográficas e socioeconômicas no TDR em português brasileiro de normo-ouvintes.

## MÉTODO

Este estudo transversal, prospectivo e multicêntrico foi aprovado pelo Comitê de Ética em Pesquisa do Hospital Universitário Onofre Lopes (nº 2525183). Todos os participantes ou responsáveis assinaram o Termo de Consentimento ou Assentimento Livre e Esclarecido (TCLE), e os sujeitos entre 12 a 18 anos assinaram o Termo de Assentimento.

A amostra deste estudo foi composta por 151 sujeitos normo-ouvintes com idade entre 12 a 79 anos (34,66±16,17). Participaram deste estudo sujeitos do Rio Grande do Norte e da Paraíba, que realizaram audiometria tonal liminar, timpanometria e o teste de dígitos no ruído em sequência no mesmo dia. Os sujeitos da Paraíba foram recrutados e avaliados no Hospital Universitário da Universidade Federal da Paraíba, enquanto os do Rio Grande do Norte compareceram a um Serviço de Saúde Auditiva credenciado ao Sistema Único de Saúde por meio de amostras de conveniência entre setembro de 2018 e março de 2020. As equipes envolvidas na aplicação do TDR e realização da audiometria tonal liminar em ambos os locais foram treinados e seguiram os mesmos procedimentos de coleta, estando envolvidos num estudo Multicêntrico entre a a Universidade Federal do Rio Grande do Norte, Universidade Federal da Paraíba, Faculdade de Odontologia de Bauru da Universidade de São Paulo, Universidade de Pretória e empresa *Hear X* da África do Sul. Os sujeitos foram recrutados pelos pesquisadores na sala de espera do Serviço de Saúde Auditiva.

Participaram do estudo aqueles que se encaixavam no critério de inclusão: sujeitos com idade maior ou igual a 12 anos, que tivessem realizado audiometria tonal liminar, que pudessem identificar a representação gráfica dos dígitos de 0 a 9 e que não apresentassem alguma deficiência motora ou visual não corrigida, bem como déficit cognitivo ou distúrbios neurológicos conhecidos, além de apresentar média quadritonal até 25 dB NA na audiometria tonal liminar bilateralmente, sem alterações de orelha média confirmada pela presença de timpanometria com pico de pressão entre +100 a -100daPA e complacência maior do que 0,3 cc. Este foi o critério adotado para considerar os sujeitos como normo-ouvintes.

Para a realização do TDR foi utilizado um *smartphone* Motorola Z3 play com acesso à internet da versão teste do TDR. Esta versão foi desenvolvida para a realização das pesquisas no Brasil, sendo uma versão preliminar do aplicativo. Foram utilizados fones intra-auriculares originais do *smartphone* para o procedimento com adaptador original para conectar os fones ao celular. Estudo preliminar não evidenciou diferença limiar de reconhecimento de dígitos no ruído (LRF) do TDR entre os fones intra-auriculares e o TDH-39^(10)^. Os pesquisadores realizaram o registro inicial dos sujeitos na versão teste do TDR. Em ambiente silencioso, porém não acusticamente tratado, todos os sujeitos foram orientados a escutarem sequências de três dígitos em meio a um ruído branco apresentado simultaneamente e clicarem os números que ouviriam na tela do *smartphone*. Caso o sujeito não escutasse algum dos dígitos, era orientado a supor uma sequência de dígitos para dar sequência ao teste. Após a instrução e antes do início do teste, os sujeitos eram informados que poderiam ajustar o volume do teste na opção própria da versão preliminar do aplicativo, para garantir que escutassem os números de maneira clara e confortável. Esse foi o único ajuste de volume, realizado pelo próprio usuário como também ocorre na versão final do aplicativo disponibilizada em outros idiomas, estando o volume do dispositivo fixo no máximo.

O teste envolveu apresentação aleatória de 23 sequências de três dígitos (0-9) no ruído branco a 70 dB SPL numa relação sinal-ruído fixada entre -20dB a 20dB de modo diótico (*inphase*). As sequências foram apresentadas inicialmente numa relação sinal/ruído de 0dB e variaram conforme respostas do paciente sem interferência do pesquisador. As três primeiras apresentações de cada estímulo foram desconsideradas do resultado final, atuando como momento de treinamento. Os sujeitos foram orientados a selecionar no teclado virtual do *smartphone*, a sequência de três dígitos que havia escutado junto ao ruído. Em caso de acerto, o sinal de fala seguinte e o ruído apresentavam uma relação sinal ruído menor. Contudo, caso o sujeito errasse a sequência, o software iria aumentar a relação sinal ruído na próxima sequência. Essa variação foi de 2 decibéis em ambos os casos. Após as 23 sequências, era gerado pelo software o limiar de reconhecimento de dígitos no ruído (*LRF*) pela média da relação sinal ruído entre as sequências 4 e 23. Na tela final era mostrado um número, que representa o valor do LRF. Além disso, foi realizado o *download* em uma tabela, com os resultados detalhados de cada apresentação por sujeito. Estas informações foram tabuladas em banco de dados no Excel. Espera-se em normo-ouvintes valores do LRF do TDR mais negativos que significam melhor desempenho no teste.

Para determinar o status audiológico foi realizada a audiometria tonal liminar em cabina acústica com audiômetro calibrado do modelo AD229e. As frequências testadas foram de 250Hz a 8000Hz em via aérea e de 500Hz a 4000Hz em via óssea caso paciente apresentasse resposta mínima em alguma destas frequências em intensidade superior a 25 dB NA no teste de via aérea. Foram classificados como normo-ouvintes aqueles que apresentaram limiares auditivos da média quadritonal (500 HZ, 1000 Hz, 2000 Hz e 4000 Hz) até 25 dB NA em ambas as orelhas^(19)^.

Os dados foram coletados de forma independente, tanto pelos fonoaudiólogos que realizaram a audiometría tonal liminar e como pelos que aplicaram o TDR. O resultado de cada procedimento foi conhecido pela equipe apenas na tabulação dos dados.

Além dos testes audiológicos, todos os sujeitos responderam oralmente a um questionário para a coleta das seguintes variáveis demográficas: idade e sexo; assim como as variáveis socioeconômicas: escolaridade e nível socioeconômico.

Em relação a idade, a amostra foi subdividida nas seguintes faixas-etárias: G1:12-19 anos, G2:20 a 39 anos, G3:40 a 59 anos e G4: 60 a 79 anos.

A escolaridade foi categorizada em três níveis: baixa, para sujeitos que se declararam analfabetos ou tendo cursado o ensino fundamental incompleto, correspondendo ao tempo de 0 a 9 anos de escolaridade; média, para sujeitos que cursaram do ensino fundamental completo ao ensino médio completo, equivalente a 9 a 16 anos de escolaridade, e escolaridade alta para aqueles que cursaram ensino superior incompleto ou completo, com mais de 16 anos de escolaridade.

Quanto ao nível socioeconômico foi aplicado o questionário da Associação Brasileira de Empresas de Pesquisa (ABEP)^(20)^. O documento é dividido em duas categorias de informações. Na primeira o sujeito informa sobre os itens que possui em casa e suas respectivas quantidades; os itens incluem banheiro, microcomputador, máquina de lavar louça, automóvel, micro-ondas etc. A segunda categoria identifica o grau de instrução do chefe de família (relativo à escolaridade) e o acesso a serviços públicos (água encanada e rua pavimentada). A partir da análise proposta pela ABEP^(20)^ foi realizada a categorização, neste estudo, em três níveis socioeconômicos: baixo, com pontuação de 1 a 16 pontos; médio para sujeitos com pontuação de 17 a 28 pontos; alto para os sujeitos que pontuaram de 29 a 100 pontos.

Na análise dos dados foi verificada a normalidade da distribuição, aplicando do Teste de Kolmogorov-Smirnov. Ao não ser constatada distribuição normal, foram utilizados os testes não-paramétricos Kruskal-Wallis e Mann-Whitney na comparação entre as variáveis independentes (sexo, faixa-etária, escolaridade e nível socioeconômico) em relação a variável dependente que foi o valor do LRF do TDR. Foi adotado o nível de significância de 5%.

## RESULTADOS

A amostra deste estudo foi composta por 151 sujeitos normo-ouvintes com idade entre 12 a 79 anos (34,66±16,17). O LRF do TDR da amostra total de sujeitos normo-ouvintes teve a mediana de -9,6 dB, com valor mínimo -13 dB e o valor máximo de +1,8 dB do LRF do TDR.

A Tabela 1 apresenta a distribuição da amostra em função das variáveis demográficas e socioeconômicas, bem com a estatística descritiva e inferencial do LRF do TDR em função delas.

**Tabela 1:** Estatística descritiva e inferencial do LRF do TDR em função das variáveis demográficas e socioeconômicas da amostra estudada

		**LRF do TDR diótico**							**p – valor**
		**N (%)**	**Média (dp)**	**Min**	**Max**	**Q25**	**Med**	**Q75**	
Sexo	Masculino	42 (27,82%)	-9,13 (2,84)	-13,00	-1,00	-11,20	-9,80	-7,40	,404
	Feminino	109 (72,18%)	-8,52 (3,512)	-12,60	5,20	-11,10	-9,40	-7,40	
Faixa Etária	12-19 (G1)	15 (9,94%)	-8,86 (3,87)	-12,60	1,80	-11,60	-10,20	-7,40	**
	20-39 (G2)	83 (54,97%)	-9,71 (2,33)	-13,00	-2,00	--11,40	-8,40	-6,68	
	40-59 (G3)	41 (27,15%)	-7,34 (3,91)	-11,60	5,20	-10,50	-8,00	-5,60	
	60-79 (G4)	12 (7,94%)	-6,00 (3,78)	-10,20	2,00	-8,40	-8,00	-3,20	
Escolaridade	Baixo	31 (20,53%)	-6,26 (4,17)	-11,40	3,60	-9,20	-7,40	-3,60	,001*
	Médio	38 (25,17%)	-8,47 (3,24)	-11,80	5,20	-10,80	-9,20	-7,40	
	Alto	82 (54,30%)	-9,71 (2,47)	-13,00	-2,00	-11.60	-10,20	-8,40	
NSE	Baixo	12 (7,94%)	-4,83 (5,04)	-10,80	3,6	-8,90	-6,10	0,20	,001*
	Médio	44 (29,14%)	-7,79 (3,53)	-11,80	5,2	-10,50	-8,50	-6,50	
	Alto	95 (62,92%)	-9,59 (2,45)	-13,00	--2,00	-11,40	-10,20	-8,20	

* ρ-valor <0.05 significante. Testes de Kruskal Wallis e Mann-Whitney;

** p-valor >0,05 (grupos G1XG2, G1XG3 e G3XG4) e p<0,05 (G1XG4, G2XG3, G2XG4);

N: Número de sujeitos (%); **Legenda:** LRF – limiar de reconhecimento de dígitos no ruído. Q25 –quartil 25%. Q75 – quartil 75%. N = N amostral; % = porcentagem. NSE – Nível Socioeconômico

Observa-se que todas as variáveis, menos a variável sexo, influenciaram no resultado do LRF do TDR. Em relação à faixa-etária, o grupo composto pelos mais jovens (12 a 19 anos) teve o LRF do TDR significativamente melhor apenas quando comparado aos dos idosos (60 a 79 anos). Já os adultos entre 20 a 39 anos tiveram o LRF do TDR melhores quando comparados aos adultos entre 40 e 59 anos e aos idosos acima de 60 anos.

## DISCUSSÃO

Os resultados deste estudo demonstraram que o LRF do TDR em português Brasileiro em sujeitos normo-ouvintes apresenta associação com a escolaridade e nível socioeconômico, bem como em relação à faixa-etária. Não houve evidências de influências do sexo no LRF do TDR.

### LRF do TDR em normo-ouvintes

A média de -8,47 dB no LRF do TDR em condição diótica (*inphase*) encontrada entre os sujeitos normo-ouvintes deste estudo está maior do que a média referida por Potgieter et al.^(7)^ também em sujeitos normo-ouvintes (-10,7dB, com referência das duas orelhas < 15 dB de média quadritonal), porém assemelha-se ao valor do ponto de corte encontrado por estes autores de -8,4 dB pela melhor orelha ou -8,9 dB para ambas as orelhas com audição normal (<15dB de média quadritonal).

Os resultados esperados assemelham-se a média do LRF do TDR dos sujeitos sem competência no Inglês (-8,7 dB)^(11)^, visto que aqueles com maior competência no Inglês ou nativos tiveram a média do LRF do TDR em -10,4 e -10,2 dB, respectivamente.

A avaliação do teste de dígitos se mostrou confiável em grupos normo-ouvintes, assim como em grupos usuários de aparelhos auditivos e de implante coclear, ressaltando não só a sua capacidade de ferramenta de rastreio auditivo como de avaliação do processo de reabilitação^(20,21)^, assim como identificado na presente amostra. Contudo, pela influência das variáveis socioeconômicas e demográficas, o resultado do LRF não foi semelhante aos achados da versão teste de forma integral.

### Sexo

A amostra estudada foi composta por 72,18% por sujeitos do sexo feminino, embora tenha tido esta predominância sobre os sujeitos do sexo masculino não foi evidenciada diferença entre os resultados do LRF do TDR entre os sexos. Esta variável não se mostrou preditora dos resultados do LRF do TDR em estudo com o TDR em inglês com diferentes competências no inglês e falantes de outras línguas da África do Sul^(11)^.

### Idade

Sujeitos com idade acima de 60 anos apresentaram LRF do TDR diótico pior quando comparados aos mais jovens (12-39 anos). Este resultado difere de outro estudo que demonstrou resultados de LRF semelhantes aos de jovens a partir dos 12 anos de idade^(22)^. Achado semelhante a este estudo também foi evidenciado no estudo com o TDR em português Brasileiro sendo utilizado com crianças normo-ouvintes e sem transtornos do processamento auditivo e com transtorno do processamento auditivo entre 8 a 11 anos^(13)^. Os valores do LRF destes dois grupos tiveram médias similares aos encontrados em outra pesquisa do Teste de dígitos no ruído em português brasileiro^(12)^. Esses achados podem indicar o impacto das diferenças sociodemográficas próprias da amostra, já que apesar de terem faixas etárias diferentes, esses estudos têm população originária da mesma região.

Um estudo retrospectivo com 24.072 sujeitos constatou que a idade é um fator de influência nos resultados do TDR. Fator que pode ser explicado devido a diminuição da cognição nos idosos e a dificuldade na compreensão das explicações dadas^(23)^, na qual essas dificuldades, mesmo sendo mínimas, tornam-se um desafio e um transtorno na interação da população dessa faixa etária com o *smartphone* ^(24)^. Pesquisadores abordaram sobre a necessidade de considerar a idade ao determinar o resultado do teste, pois a precisão do resultado pode diferenciar dependendo da faixa etária. Esses autores evidenciaram que a idade pode ser um preditor significativo do LRF do TDR para ouvintes com melhor orelha MQ≤25 dB NA^(25)^.

Informações semelhantes a estas podem ser constatadas no estudo no qual se observou declínio de aspectos cognitivos e da capacidade do processamento auditivo com outras tarefas de reconhecimento de fala no ruído conforme o avanço da idade^(26)^.

Desta forma, é importante considerar a idade ao determinar o resultado do TDR em sujeitos normo-ouvintes, pois isso pode contribuir para a precisão do resultado do teste de triagem^(27)^. Além disso, é possível que outros estudos considerem a aplicação de um rastreio cognitivo, para identificar o impacto dessas possíveis alterações no resultado do TDR.

### Escolaridade

Apesar de uma predominância de sujeitos com escolaridade alta, foi possível observar que sujeitos com maior escolaridade tendem a ter melhor compreensão de fala no ruído, com melhores LRF.

Outros pesquisadores também observaram que a diferença de escolaridade está associada a dificuldades auditivas para atividades de processamento auditivo. Entre as hipóteses levantadas está a diferença de histórico da população idosa que teve acesso precário à educação^(27)^.

Dentre as atividades de processamento auditivo, a compreensão de fala no ruído também é prejudicada com menor índice de escolaridade. Abre-se a discussão se há nesse resultado influência da memória de trabalho além dos fatores auditivos, embora essa característica tenha se mostrado significante em outras habilidades do processamento auditivo, mas não no teste de fala no ruído^(28)^.

### Nível socioeconômico

O nível socioeconômico é um parâmetro que leva em consideração escolaridade, ocupação e renda^(17)^. Portanto, as variáveis socioeconômica e de escolaridade abordadas neste estudo estão diretamente relacionadas. É possível observar essa equivalência na análise estatística do estudo, em que há diferença estatisticamente significante entre os grupos baixos, médio e altos tanto na escolaridade quanto no nível socioeconômico.

Assim, quanto mais elevado o nível socioeconômico e a escolaridade mais negativo o valor do LRF do TDR.

O nível socioeconômico está intrinsecamente ligado à saúde, de modo que aqueles com melhor poder aquisitivo normalmente são mais saudáveis do que aqueles com o nível socioeconômico mais baixo^(29)^. Corroborando com isso, constata-se que dentre a população mundial que apresenta perda auditiva, onde há maior prevalência em países de baixa e média renda, ainda há baixa capacidade de atendimento dos serviços de saúde^(1)^, mostrando que a prevalência dessa enfermidade varia de acordo com a região^(30)^ e que o componente socioeconômico é um dos fatores determinantes para o seu surgimento.

Os fatores socioeconômicos e de escolaridade podem se relacionar com o status audiológico de um indivíduo. Um estudo realizado com 3379 pessoas nos Estados Unidos, mostrou que há correlação direta do nível socioeconômico e escolaridade com a perda de audição, no qual essa deficiência pode ser um fator ou um produto da caracterização socioeconômica do sujeito. Além disso, a pesquisa revelou associações entre desemprego, idade, escolaridade, sexo e estado auditivo. No caso do TDR, a situação que se encontrou foi que, aqueles indivíduos que possuíam a audição normal (comprovada pela audiometria tonal), de modo geral, não tiveram um desempenho adequado para seu status audiológico^(16)^.

Concomitante à utilização de métodos de promoção e prevenção à saúde através de tecnologias digitais, embasar conhecimentos acerca da influência da idade no TDR, em principal na população idosa, deve ser constante devido às restrições físicas e a própria dificuldade de manipulação do equipamento nessa população. Atrelado a isso, há a probabilidade do TDR ter como alvo a população mais jovem, onde a prevalência da perda auditiva ainda é menor^(23)^, mas que vem sendo a preocupação mundial visto o uso excessivo de dispositivos individuais de música utilizados com intensidade excessiva.

Portanto, o ideal seria considerar os diferentes fatores estudados no resultado do TDR, adotando-se diferentes pontos de cortes de acordo com os níveis socioeconômicos, de escolaridade ou de idade que o indivíduo se encontre.

### Limitações do estudo

Ao analisar pontos que podem ter alterado o resultado da pesquisa, destaca-se a versão de teste disponibilizada utilizada para teste de dígitos no ruído. Sendo o acesso deste a partir de um link da internet, inviabilizando sua realização sem rede de dados. Tendo uma resposta ao toque da tela ainda em desenvolvimento que, por vezes, necessitava de intervenção do pesquisador para correção de zoom que se ativou a um rápido toque duplo do sujeito no dispositivo. A experiência de uso para o sujeito poderia ser melhorada por meio da versão finalizada do aplicativo.

Outras categorizações de faixa-etária, escolaridade e nível socioeconômico poderiam alterar os resultados encontrados, assim como as características da população do nordeste, sendo sugerida ainda a realização de outros estudos em outras regiões brasileiras.

### Perspectivas para as futuras pesquisas

Estudos posteriores devem ser conduzidos para estudar a versão finalizada do aplicativo em comparação com a versão teste. Além disso, devem ser realizadas futuras pesquisas que considerem a análise das variáveis demográficas e socioeconômicas estudadas no ponto de corte do teste, a fim de alcançar uma melhor acurácia no teste de dígitos no ruído em português considerando as características populacionais, e favorecendo sua aplicação em larga escala como instrumento de identificação da perda auditiva.

## CONCLUSÃO

O teste de dígitos no ruído diótico no Português Brasileiro tem associação com variáveis extrínsecas ao teste, apresentando melhores respostas entre 20 e 39 anos, com nível socioeconômico alto e escolaridade alta. Não foi observada influência do sexo no reconhecimento dos dígitos do TDR.
