# Supplementary material for: ERRATUM: Digits-in-noise test in Brazilian Portuguese: how demographic and socioeconomic variables influence normal-hearing subjects
Source: Codas. 2022 Jun 27;34(6):e20220110. doi: 10.1590/2317-1782/20212022110 (PMC9886287; doi:10.1590/2317-1782/20212022110)
Supplement: Translation

# Translation: ERRATA: Teste de dígitos no ruído no Português Brasileiro: influência das variáveis demográficas e socioeconômicas em normo-ouvintesa

Devido a problemas técnicos durante a produção editorial do artigo “Teste de dígitos no ruído no Português Brasileiro: influência das variáveis demográficas e socioeconômicas em normo-ouvintes” (DOI https://doi.org/10.1590/2317-1782/20212021274), publicado em CoDAS, 2022;34(6):e20210274, foi publicado com um erro que omitiu o texto de agradecimentos do artigo.

Na página 6, onde o texto termina e inicia-se a lista de referências bibliográficas, leia-se:

**AGRADECIMENTOS**

Os autores agradecem as contribuições dos pesquisadores De Wet Swanepoel e Cas Smits pela parceria no desenvolvimento do teste de dígitos no ruído na versão em Português Brasileiro.

A prestadora de serviços editoriais pede desculpas pelos erros.
